# Paris Crime

**Published:** 1882-01

**Authors:** 


### PARIS CRIME.

The other night two young clerks were playing billiards in a café of Boulevard St. Germain, and talked freely of their concerns without heeding a man who seemed asleep at an adjoining table. One of them said to the other:

"I have just inherited four hundred dollars."

"O, then, we are going to have a jolly time of it."

"No, no! I will not touch one cent of that money. It is still in my lodgings, safely hid in a chest of drawers under my shirts. To-morrow I am going to carry it to the stockbroker, and buy threes."

A few minutes afterward the sleeping man woke, arose, took his hat and went out. An hour afterward the clerks paid for what they had taken, took their hats; one of them—he who had inherited four hundred dollars—found that his hat had been taken, probably by the sleeper, and another left in its place. As the hat left was just as good as the hat taken, and as the former fitted him quite as well, he bore the exchange philosophically, and jogged home. He had no sooner entered his lodgings than he saw they had been entered with false keys; his chest of drawers broken open, and his four hundred dollars stolen. He did not sleep that night. The hat left him did not altogether suit him, so he went to his hatter to change it. His hatter told him that the evening before a man had come to the shop, and had said that he had inadvertently taken a gentleman's hat, and seeing the latter's name inside, he had called, thinking the hatter might be able to give him the owner's name and address, that he might restore the hat accidentally taken. The hatter gave the name and address. The burglar secured four hundred dollars by the information given. The burglar's hat was left with the hatter, and a new hat selected. After the clerk left, the hatter took out the lining of the burglar's hat, and, to his surprise, found under it a letter, bearing an address. He read it, and found it was from a burglar, promising aid in a contemplated burglary which had been planned by the person to whom the letter was addressed. The hatter took the letter to the police, and told what had occurred. Before sunset both burglars were arrested.

